# The Latest Data Concerning the Etiology and Pathogenesis of Irritable Bowel Syndrome

**DOI:** 10.3390/jcm13175124

**Published:** 2024-08-29

**Authors:** Vlad Alexandru Ionescu, Gina Gheorghe, Teodor Florin Georgescu, Nicolae Bacalbasa, Florentina Gheorghe, Camelia Cristina Diaconu

**Affiliations:** 1 Faculty of Medicine, University of Medicine and Pharmacy Carol Davila Bucharest, 050474 Bucharest, Romania; vladalexandru.ionescu92@gmail.com (V.A.I.); florin.georgescu1@yahoo.com (T.F.G.); 2 Internal Medicine Department, Clinical Emergency Hospital of Bucharest, 105402 Bucharest, Romania; 3 General Surgery Department, Clinical Emergency Hospital of Bucharest, 105402 Bucharest, Romania; 4 Department of Visceral Surgery, Center of Excellence in Translational Medicine, Fundeni Clinical Institute, 022328 Bucharest, Romania; nicolae.bacalbasa@umfcd.ro; 5 Department of Biochemistry, Synevo Romania, 077040 Chiajna, Romania; florentina.9412@gmail.com; 6 Academy of Romanian Scientists, 050085 Bucharest, Romania

**Keywords:** irritable bowel syndrome, pathophysiology, gut–brain axis, dysbiosis, low-grade inflammation

## Abstract

Globally, irritable bowel syndrome (IBS) is present in approximately 10% of the population. While this condition does not pose a risk of complications, it has a substantial impact on the patient’s quality of life. Moreover, this disease has a significant financial impact on healthcare systems. This includes the direct costs associated with the diagnosis and treatment of these patients, as well as the indirect costs that arise from work absenteeism and reduced productivity. In light of these data, recent research has focused on elucidating the pathophysiological basis of this condition in order to improve the quality of life for affected individuals. Despite extensive research to date, we still do not fully understand the precise mechanisms underlying IBS. Numerous studies have demonstrated the involvement of the gut–brain axis, visceral hypersensitivity, gastrointestinal dysmotility, gut microbiota dysbiosis, food allergies and intolerances, low-grade mucosal inflammation, genetic factors, and psychosocial factors. The acquisition of new data is crucial for the advancement of optimal therapeutic approaches aimed at enhancing the general health of these patients while simultaneously reducing the financial burden associated with this ailment.

## 1. Introduction

The disorders of gut–brain interactions (DGBIs), commonly referred to as gut–brain axis diseases, have a global prevalence of roughly 40% [1]. Two-thirds of these patients present a chronic, fluctuating clinical evolution, characterized by intermittent periods of remission interspersed with episodes of symptomatology aggravation [1,2]. Gastrointestinal functional disorders are conditions with a complex, incompletely understood pathophysiology that account for at least one-third of presentations to gastroenterology clinics [1,2].

The most common symptoms of gastrointestinal diseases are abdominal pain, nausea, vomiting, flatulence, postprandial fullness, diarrhea, and constipation [3]. These symptoms are prevalent in an extensive array of organic disorders, including gastroparesis, celiac disease, inflammatory bowel disease, gastrointestinal neoplasia, and peptic disease [1,3]. Nevertheless, it is well acknowledged that a significant proportion of patients do not exhibit any structural abnormalities that can account for their symptoms during paraclinical examinations. In such instances, a diagnosis of functional disease is made [1,3]. To enhance the scientific accuracy and mitigate the negative connotations linked to the functional term, these conditions have been reclassified as disorders of gut–brain interactions [1,3]. Current therapeutic approaches aim to address not only the primary gastrointestinal symptoms but also the underlying pathophysiological mechanisms.

In the last three decades, the Rome Foundation, comprising gastroenterologists and researchers specializing in gastrointestinal pathology, has developed and revised standardized approaches for the identification and classification of functional digestive disorders. These methods have been established through a collaborative effort involving expert consensus and the utilization of existing research [1,2]. The diagnostic criteria established by the Rome Foundation in 1990 have been modified thrice since their inception; they now define each condition according to a collection of symptoms and restricted paraclinical examinations [4]. The most recent consensus, Rome IV, adopted in 2016, classifies functional digestive diseases into 8 primary classes and 32 illnesses based on the afflicted segment [3,4,5].

Although not considered life-threatening, disorders of gut–brain interaction (DGBI) exert a substantial detrimental influence on the quality of life of patients and impose substantial financial burdens on healthcare systems [6,7]. In a recent study, Mahon et al. found that the annual expenses for managing patients with DGBI in England amounted to over GBP 72.3 million [7]. Out of this amount, approximately GBP 50 million were specifically allocated to the treatment of these patients, whether they received outpatient or inpatient care [7]. An estimated EUR 700–1600 are spent annually on the management of patients with irritable bowel syndrome (IBS) in Europe according to Farzai et al. [8]. The expenses encompass not only the direct expenses associated with diagnostic investigations and the requisite treatment for these patients, but also the broader indirect costs stemming from workplace absenteeism and diminished productivity [8]. In another study that followed 73,076 adults from 33 countries and 6 continents, 40.3% of the subjects (95% CI: 39.9–40.7) were found to have at least one DGBI [2]. In addition, the investigators noted a greater occurrence of functional dysfunction in women as opposed to men [2]. These conditions have been linked to a diminished quality of life and increased frequency of medical visits [2,9].

Functional dyspepsia, functional constipation, and IBS are among the most prevalent DGBIs [10,11]. Regarding the geographical dispersion of prevalence rates, epidemiological investigations have documented discrepancies among countries [11]. These variations can be accounted for by differences in research methodology, genetic profiles, dietary customs, or lifestyles [11].

The aim of our review is to provide the most recent information regarding the etiology and pathogenesis of IBS. We consider the understanding of this data to be crucial for the management of these patients and the enhancement of their quality of life.

## 2. Methods

In order to carry out this review, we employed recent articles that concentrated on the etiology and pathogenesis of IBS as sources of information. The search terms that we employed were “irritable bowel syndrome”, “pathogenesis”, and “etiology”. The articles were selected based on the quality of the research, which was assessed based on the size of the study group and the methodology employed, as well as the date of publication. We first defined the diagnostic principles of IBS in order to avoid any confusion with other DGBIs. In the subsequent section, we highlighted the most critical pathogenic linkages of IBS in order to subsequently delve into the evidence that implicates them in the pathogenesis of IBS.

## 3. Irritable Bowel Syndrome: Principles of Diagnosis

IBS is one of the most common DGBIs. The current data indicate that the worldwide occurrence of IBS is estimated to be around 10% when employing the Rome III criteria for diagnosis and 4% when employing the Rome IV criteria, with significant differences observed across different regions [2,12]. A disparity in prevalence based on gender has been identified, with women having a prevalence of approximately 12% and men having a prevalence of approximately 9% [2,12,13]. Another noteworthy element pertains to the overlap between IBS and various other conditions, including functional dyspepsia, depressive syndrome, anxiety disorders, somatization disorder, and gastroesophageal reflux disorder [14,15,16,17].

The diagnosis of IBS is established by examining the connection between chronic abdominal pain and intestinal motility dysfunction [18,19]. An individual in good health often has a frequency of three stools per day to three stools per week. The Bristol scale is utilized to evaluate the stool consistency. This categorizes stools into seven distinct types: normal for types 3–5; constipation for types 1 and 2; diarrhea for types 6 and 7 [20].

The primary assessment of a patient with suspicion of IBS includes a meticulous review of their medical history and a comprehensive clinical examination. Furthermore, the clinical examination should include a digital rectal examination for patients with IBS-C. It has the potential to emphasize potential functional defecation disorders while simultaneously contributing to understanding the physiopathological mechanisms that underlie the symptoms [21]. These findings play a dual role in confirming a positive diagnosis of intestinal functional dysfunction and distinguishing it from other diseases that present similar symptoms [22]. In a family history evaluation, the presence of celiac disease, colorectal malignancy, or inflammatory bowel disease should be evaluated. The physical examination of patients with IBS is typically normal. Nevertheless, mild abdominal tenderness may occasionally be detected upon palpation [22].

The diagnosis and classification of IBS are determined by applying the Rome IV Criteria, which were established in 2016 (Table 1) [5].

Paraclinically, there is no marker for IBS [23]. Laboratory tests are usually conducted to exclude the presence of an organic condition. Therefore, it is necessary to conduct the following diagnostic tests in all individuals who are suspected to have IBS: full blood count, inflammatory markers such as C-reactive protein and erythrocyte sedimentation rate, IgA anti-tissue transglutaminase antibodies and total IgA, thyroid-stimulating hormone (TSH), and in patients with diarrhea, fecal calprotectine [23,24]. All of these examinations in IBS fall within the normal range.

Further investigations are necessary when patients do not respond to first-line treatment or alarm signals are observed, such as weight loss, rectal bleeding, anemic syndrome, or palpation of an abdominal mass:

o An upper gastrointestinal endoscopy with duodenal biopsies is performed to exclude the possibility of celiac disease.
o Colonoscopy with intubation of the ileo-cecal valve, evaluation of the last few centimeters of the terminal ileum, and staged colonic biopsies (to rule out malignant pathology, microscopic colitis, or inflammatory bowel diseases).
o Fecal elastase (to exclude exocrine pancreatic insufficiency).
o Breath tests for lactose or fructose intolerance (to rule out malabsorption syndromes).
o Glucose breath test (to rule out intestinal bacterial overpopulation syndrome).
o 75-selenium homocholic acid taurine (SeHCAT) test or serum 7α-hydroxy-4 cholesten-3-one (C4), along with fibroblast growth factor 19 (FGF19) (when SeHCAT is not available), can be used in patients with diarrhea to rule out bile acid malabsorption (BAM).
o Anorectal physiology tests are required in patients with constipation who do not respond to first-line treatment [23,24,25,26,27,28].

In conclusion, the diagnosis of IBS should be made through a positive diagnostic approach rather than an exclusion diagnosis. In the absence of alarm signs/symptoms or refractory symptoms, a minimal number of examinations are necessary [24,29].

## 4. Etiology and Pathogenesis of IBS

**Figure 1:**
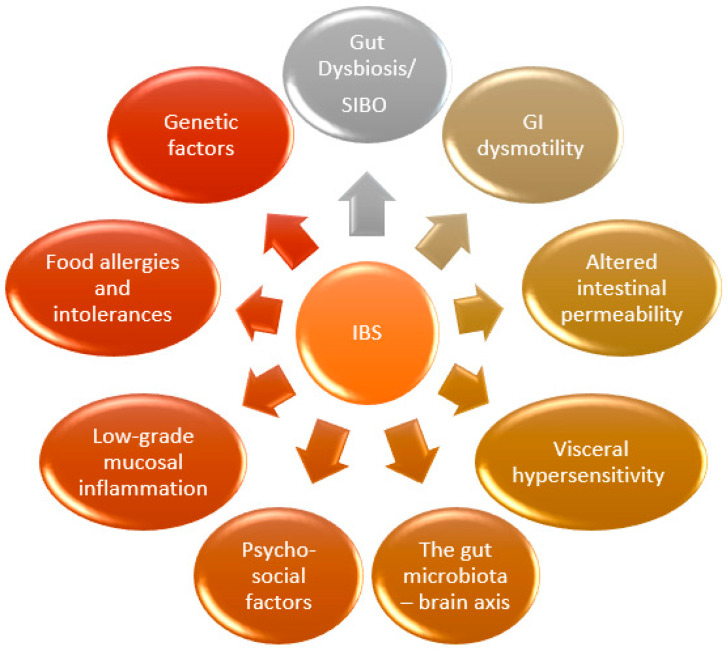
Pathophysiological mechanisms involved in the occurrence of IBS (GI—gastrointestinal; SIBO—small intestinal bacterial overgrowth).

The most important pathophysiological mechanisms involved in the development of IBS are presented in Figure 1 [30,31,32]. 

### 4.1. The Gut–Brain Axis 

The primary pathogenic mechanism involved in the development of DGBIs is disruption of the gut–brain axis [33]. The interaction between the central nervous system (CNS) and the enteric nervous system (ENS) involves intricate signal transduction systems that encompass neuronal, endocrine, immunological, and metabolic pathways [33,34,35]. There are several factors that have the potential to exert an influence on the gut–brain axis, including genetic predisposition, dietary patterns, stress levels, physical activity, cognitive function, and social interactions [33]. The gut microbiota has the capacity to synthesize a significant fraction of the neurotransmitters that have been found within the brain [36]. These neurotransmitters mostly function locally, regulating the activity of the ENS [37,38]. Nevertheless, recent research has revealed compelling data regarding the impact of neurotransmitters generated by the gut microbiome on the CNS through various mechanisms [33,39]. For example, probiotic treatment with Bifidobacteria species can enhance the production of tryptophan, a precursor to serotonin [40,41]. Additionally, certain species of lactobacilli can modify the metabolism of gamma-aminobutyric acid (GABA) and the expression of GABA receptors in the brain [40,41]. While neurotransmitters have the ability to cross the intestinal mucosal barrier, they are unable to cross the blood–brain barrier in normal circumstances [40,41,42]. An additional mechanism by which the gastrointestinal tract and brain interact is through the induction of cortisol secretion via stimulation of the hypothalamic–pituitary–adrenal (HPA) axis. The HPA axis can be activated by psychological or physical stress, leading to subsequent effects on the gut microbiota and barrier function [43]. The ultimate consequences of gut–brain axis modification are visceral hypersensitivity and gastrointestinal dysmotility.

### 4.2. Visceral Hypersensitivity

Patients with IBS often exhibit visceral hypersensitivity, which refers to a heightened reactivity to mechanical or chemical stimuli [8,44]. The perception of functional abdominal pain is mediated by the activation of specific mechanoreceptors or chemoreceptors situated in the intestinal mucosa [8,44]. The signal is sent through afferent neural pathways from these receptors to the dorsal horn of the spinal cord and ultimately to the CNS [8,44]. The selective hypersensitivity of afferent visceral nerves, which initiate at the intestinal level, in response to gas distention, has been the subject of numerous studies as a potential explanation for the symptoms observed in patients with IBS [8,44,45]. Bouin et al. assessed the sensitivity, specificity, and predictive value of pain thresholds for rectal distension in a cohort consisting of 86 patients diagnosed with IBS, 26 patients with functional constipation, 21 patients with functional dyspepsia, 31 patients with various gastrointestinal diseases, and 25 control subjects [46]. The researchers used an electric barostat for the evaluation. The authors of this study established that patients with IBS had lower pain thresholds for rectal distension than patients with other gastrointestinal disorders or control subjects [46]. In a recent study conducted by Grabauskas et al., biopsy samples obtained from the colon of individuals with IBS and a preponderance of diarrhea were examined [47]. The researchers observed a significant upregulation of cyclooxygenase-2 gene expression and elevated levels of prostaglandin E2 [47]. Furthermore, the feces of these patients were shown to contain elevated levels of tryptase, histamine, and other bioactive compounds [47]. The authors of this study reached the conclusion that the production of prostaglandin E2 by mast cells in the colon leads to the activation of EP2 receptors in the submucosal sensory fibers of neurons in the dorsal root ganglia. This activation subsequently results in the transmission of nociceptive signals to the spinal cord [47]. This mechanism was found to be essential in the induction of visceral hypersensitivity [47]. Furthermore, there are several more factors that may contribute to visceral hyperalgesia, including particular gastrointestinal mediators such as serotonin, as well as increased spinal cord excitability resulting from activation of *N*-methyl-D-aspartate receptors [8]. Serotonin production initiates with the conversion of L-tryptophan into 5-hydroxytryptophan (5-HTP) by the action of the enzyme tryptophan hydroxylase (TPH) [48]. Then, aromatic amino acid decarboxylase converts 5-HTP into serotonin (5-HT). Additionally, there are two distinct forms of TPH, namely TPH1 and TPH2. TPH accounts for 90% of the serotonin production in the intestines, specifically in enterochromaffin cells. The other 10% of serotonin synthesis is facilitated by TPH2, which is localized in the central and enteric nervous systems [48]. Previous studies have demonstrated the involvement of serotonin in various biological processes, including neurogenesis, epithelial growth, inflammation, secretion, perception, and motility [48]. The activity of serotonin on the 5HT3 receptor, which is found on sensory and myenteric neurons, leads to an increase in intestinal peristalsis and propulsive contraction [48,49]. Additional research indicated that the hormone serotonin 5-hydroxytryptamine (5-HT) may also influence visceral perception [48]. It is hypothesized that the 5-HT3 and 5-HT4 receptors may be crucial in the transmission of visceral sensations from the intestines [48]. In another recent study, Chang et al. assessed the visceromotor responses to colorectal distension in two murine models [50]. The authors showed that there is a direct proportional link between visceral hypersensitivity and the density of nerve fibers in the intestinal mucosa that express 5-HT7 [50]. They also observed a positive feedback loop between the serotonin and neurotrophin pathways through 5-HT7 activation via nerve fiber elongation [50]. Previous studies have demonstrated that the synthesis of serotonin is influenced by the composition of the gut microbiota, namely the clostridial class belonging to the Firmicutes phylum [51]. Tachykinin, a molecule that acts as an agonist for neurokinin 2 (NK2) and neurokinin 3 (NK3), induces the release of serotonin through its interaction with receptors of NK2 or NK3. The NK3 receptor is located in myenteric plexus neurons and plays an important role in inflammation [52]. The patients diagnosed with IBS have elevated levels of serotonin, suggesting that it may play a role in the development of digestive symptoms [52].

Furthermore, research employing functional magnetic resonance identified a reduction in the thickness of the insular and cingulate cortices, which are neural structures responsible for visceral perception, among individuals diagnosed with IBS [53,54]. Additionally, an atypical pattern of activity was observed in the cingulate, insular, and frontal cortices, as well as in the amygdala and hippocampus [54]. These findings indicate a modified functioning of the homeostatic and salience networks, as well as the autonomous nervous system, which may potentially explain the heightened vigilance and dysregulation of the hypothalamic–pituitary–adrenal (HPA) axis observed in individuals with IBS [54].

### 4.3. Gastrointestinal Dysmotility

Gastrointestinal motility is a complex process that requires the cooperation of neurons, intestinal Cajal cells, immune cells, and enteric smooth muscle [55]. The intestinal microbiota and its metabolites have the ability to interfere with the signaling pathways that involve enteric neurons, glia, and enteric muscle macrophages, hence affecting gastrointestinal motility [55]. For instance, enteric neuronal survival is facilitated by lipopolysaccharides and short-chain fatty acids, which are derived from the intestinal microbiota [55]. In addition, short-chain fatty acids regulate gastrointestinal motility by influencing the release of neurotransmitters and, secondarily, the communication between enteric neurons, smooth muscles, and muscle macrophages [55].

Another molecule that plays a significant role in the modulation of signaling pathways involving enterocytes, smooth muscles, and enteric neurons is serotonin [56]. The largest amount of serotonin in the body is found in enterochromaffin cells [56]. Serotonin stimulates both intrinsic primary afferent and extrinsic neurons to initiate peristaltic and secretory reflexes, respectively, and transmit information within the central nervous system [56].

Although intestinal motility in patients with IBS has been the subject of numerous studies, no motor activity pattern has been identified as a diagnostic indicator for this condition to date. A considerable portion of individuals diagnosed with a DGBI exhibit motor abnormalities in the gastrointestinal tract. These abnormalities include a heightened frequency and irregularity of luminal contractions, extended transit time in IBS-C, and exaggerated motor responses to cholecystokinin and food intake in IBS-D [57,58,59]. The clinical significance of these motor function alterations remains incompletely established. Nevertheless, previous studies demonstrated that the use of pharmacological agents to enhance intestinal motility in individuals suffering from IBS-C can effectively decrease abdominal flatulence and enhance symptom relief [59].

According to recent research, approximately 30% of patients with IBS-D may actually have bile acid diarrhea (BAD), and approximately 1% of the general population suffers from bile acid malabsorption (BAM) [28]. As a consequence of the increased influx of bile acids into the colon, the secretion of electrolytes and intestinal motility increase, resulting in diarrhea. Due to its chronic nature, this condition has an important negative impact on the quality of life of patients [28].

### 4.4. Gut Microbiota Dysbiosis

The available evidence indicates that there are variations in gut microbiota between healthy individuals and those diagnosed with IBS [60]. Furthermore, alterations in the composition of the intestinal microbiome have been seen in relation to the primary symptom, either diarrhea or constipation [60,61]. These theories are supported by a study that found that inoculating mice with the fecal microbiota of patients with a DGBI was associated with transferred visceral hypersensitivity [62]. Mancabelli et al. assessed the intestinal microbiome in stool samples from 147 patients with functional constipation and 68 control subjects using 16S ribosomal ribonucleic acid (rRNA)-based sequencing analysis [61]. A decrease in the presence of Bacteroides, Coprococcus 3, and Roseburia species was observed in individuals diagnosed with functional constipation compared to the group of control subjects [61]. Furthermore, the metabolic capacities of the intestinal bacteria were assessed in five patients with functional constipation and five control participants using the MiSeq platform [61]. Based on the conducted analysis, it was determined that healthy individuals have an enhanced pathway associated with carbohydrate, fatty acid, and lipid metabolism in comparison to patients diagnosed with functional constipation [61]. In contrast, it was observed that the microbiomes associated with functional constipation display an elevated expression of genes that are involved in the processes of hydrogen generation, methanogenesis, and glycerol disintegration [61].

Certain patients have presented with an infectious enteritis that occurred prior to the manifestation of symptoms that ultimately resulted in the IBS diagnosis [63]. The studies that assessed this pathogenic association documented a heightened susceptibility to the development of a DGBI following infections caused by bacteria, viruses, protozoa, or helminths [63]. In a recent study, Klem et al. found a 4.2-fold increase in the risk of developing IBS in patients who had an infectious enteritis in the preceding 12 months, and a 2.3-fold increase in those who had an infectious enteritis more than 12 months before [63]. Additionally, 13.8% of patients with enteritis caused by a bacterial infection and 41.9% of those with enteritis caused by protozoa or parasites developed IBS according to same authors [63]. A distinct meta-analysis comprising 18 trials documented a six-fold elevation in the probability of developing IBS subsequent to an acute gastrointestinal infection [64]. Post-infectious IBS risk factors include young age, female gender, prolonged febrile syndrome, and the concurrent presence of anxiety and depressive disorders [63,64].

A pathogenic association between IBS and small intestinal bacterial overgrowth (SIBO) is supported by contradictory data [65,66]. Studies demonstrating abnormal levels of hydrogen in the exhaled air of patients with IBS during the glucose breath test provide support for this association [67,68]. Furthermore, it was observed that individuals diagnosed with IBS, particularly those experiencing constipation, exhibited an elevated generation of methane, a byproduct resulting from the metabolic activities of gut bacteria [69]. Methane, functioning as a neurotransmitter, was demonstrated to have a significant role in determining the duration of transit in the ileum and colon [69]. Nevertheless, alternative research indicated that the abnormal breath tests observed in individuals diagnosed with IBS may be attributed to an accelerated oro-cecal transit rather than SIBO [70]. Moreover, it was suggested that the amelioration of symptoms following antibiotic therapy in individuals diagnosed with IBS could potentially be attributed to modifications to the gut microbiome and improvements in gut motility, rather than the presence of SIBO [71]. Thus, future studies are needed to test these hypotheses.

### 4.5. Food Intolerances

Food intolerances are estimated to affect around 5% of the general population [72]. Patients with IBS frequently perceive a direct link between consuming certain foods and aggravation of symptoms (Table 2) [72,73]. Around 20–65% of them assign digestive symptoms to food intolerances [72]. Nevertheless, a disparity exists between the subjective perception of food allergy incidence among patients with functional intestinal disorders and the real occurrence rate [72]. The worsening of symptoms following the consumption of food can be attributed to an atypical motor response of the gastrointestinal tract, an atypical eating pattern, or psychological distress, rather than being solely attributed to food intolerances [72].

The current findings indicate that nutrition has a significant role in the pathogenesis of IBS. According to one theory, fermentable monosaccharides, disaccharides, oligosaccharides, and polyols (FODMAPs) are transported to the colon, where they undergo bacterial fermentation [74,75]. This process leads to an elevation in intestinal permeability and the occurrence of low-grade mucosal inflammation [74,75]. Furthermore, recent research has indicated that FODMAPs possess osmotic activity, which can contribute to an increase in the water content of the entire gastrointestinal tract [76]. In addition, once reaching the colon, FODMAPs are readily utilized by the intestinal microbiota during the fermentation process. This leads to an increase in the formation of short-chain fatty acids, intestinal gas generation, and exacerbation of luminal distention [76]. Additionally, it has been proven that a low FODMAP diet also acts by activating Meissner’s plexus and modulating neuroenteric sensory transmission, which stimulates intestinal secretion and motility and accelerates transit time [74,75,76].

Fructose sensitivity has been proposed as a probable cause of carbohydrate malabsorption accompanied by abdominal flatulence [77,78]. The available literature provides evidence that there is a lack of significant disparities in carbohydrate absorption between those diagnosed with IBS and healthy individuals [75,76,77,78]. However, patients with DGBI exhibited a more rapid onset of abdominal flatulence subsequent to consuming these nutrients, as evidenced by carbohydrate challenge tests, when compared to healthy subjects [75,76,77,78].

An association between non-celiac gluten sensitivity and IBS has been revealed by additional research [79,80,81]. In patients with IBS-D, Wahnschaffe et al. demonstrated that elevated levels of anti-gliadin IgG antibodies and human leukocyte antigen DQ2 (HLA-DQ2) expression can predict the response to a gluten-free diet [82]. An additional recent study established a correlation between gluten consumption, heightened intestinal permeability, and frequency of gastrointestinal movements among patients who tested positive for HLA-DQ2/8 [82]. In all these cases, a thorough evaluation of the patient is required to rule out the diagnosis of celiac disease.

### 4.6. Low-Grade Mucosal Inflammation

Immunohistological studies have shown the existence of inflammatory changes in the intestinal mucosa of patients with a DGBI, particularly those with post-infectious IBS and IBS-D [84,85,86]. One of the most notable histological changes found was the presence of an inflammatory infiltration with lymphocytes in both the small intestine and colon [84,85,86]. As an example, in a study involving jejunal biopsies from patients with IBS, Törnblom et al. observed an increase in the lymphocyte count in the myenteric plexus in 90% of the patients and secondary neuronal degeneration in 60% of cases [87]. The occurrence of these events can be explained by the secretion of certain mediators (proteases, nitric oxide, or histamine) by lymphocytes, which have the ability to activate the enteric nervous system and consequently induce atypical motor responses in the intestines [87]. Recent research provides further evidence for the presence of corticotropin-releasing factor (CRF) in the eosinophils of intestinal mucosa, as well as for a correlation between this biomarker and both psychological stress and intestinal dysfunction [85]. Using transmission electron microscopy and gene/protein expression analysis, Salvo-Romero et al. analyzed the eosinophil activation profile and CRF content in biopsy samples from the jejunal mucosa of patients with diarrhea-predominant IBS and healthy subjects [85]. Based on the conducted analysis, it was shown that the patients with IBS exhibited a more prominent pattern of eosinophil degranulation in comparison to individuals in the control group [85]. Additionally, a greater concentration of CRF was detected within the cytoplasmic granules of eosinophils. The aforementioned changes were linked to the clinical severity of IBS, stress levels, and depression [85].

An elevation in serine protease activity was seen in stool tests conducted on individuals diagnosed with diarrhea-predominant IBS [88]. Moreover, increased intestinal permeability and visceral pain were observed in rodents intracolonically transplanted with fecal matter from patients whose stool analysis revealed elevated serine protease activity [89]. Other recent research documented a rise in the quantity of mast cells within the intestines of these individuals, as well as a correlation between abdominal pain and activation of these effector cells in close proximity to intestinal nerve structures [90,91].

Elevated proinflammatory cytokines were identified in the peripheral blood of patients diagnosed with IBS via proteomic analyses [92,93,94]. Furthermore, it was demonstrated that mononuclear cells in the plasma of these patients, in comparison to healthy individuals, produce higher levels of tumor necrosis factor-alpha (TNF-alpha), which has been linked to the degree of anxiety [95,96].

### 4.7. Altered Intestinal Permeability

A significant proportion of patients with IBS, particularly those with IBS-D, exhibit altered intestinal permeability [97]. Diet, microbiome alterations, mast cell activation, and the activity of mediators such as serotonin, vasoactive intestinal polypeptide, cysteine, and serine protease are among the mechanisms that underlie IBS patients’ intestinal barrier dysfunction [97]. Fritscher-Ravens et al. demonstrated in a recent study that changes in the intestinal barrier correlated with both the severity and the frequency of abdominal pain in patients with IBS [98]. Additionally, studies have correlated the ultrastructural disturbance of the tight junctions in the jejunum, colon, and rectum with both a higher frequency and a lower consistency of the stool in patients with IBS-D [99]. Researchers identified a down-regulation of the TESK1/CFL (testis-associated actin remodeling kinase/cofilin 1) pathway at the jejunal level in women with IBS-D. This pathway is involved in regulating cytoskeleton dynamics and, secondarily, intestinal movements [99]. Additional research suggested a possible relationship between impaired intestinal barrier function and psychological stress in patients diagnosed with IBS [97]. Nevertheless, the evidence that supports this hypothesis is inconclusive [97].

### 4.8. Genetic Factors

Research on genetic polymorphisms indicates that an inherited predisposition to IBS may exist [100,101]. Eijsbouts et al. compared healthy subjects to patients with IBS in a genome-wide analysis [101]. These authors identified and validated six genetic susceptibility loci for IBS [101]. The genes involved were CKAP2/TPTE2P3, NCAM1, DOCK9, CADM2, BAG6, and PHF2/FAM120A. Among them, it has been demonstrated that NCAM1, DOCK9, CADM2, and PHF2/FAM120A exhibit associations with anxiety disorders [101]. Based on these findings, Eijsbouts et al. proposed that the correlation between IBS and anxiety disorders can be explained by the presence of shared pathogenic mechanisms [101]. Other genomic investigations showed the existence of a variation in the gene encoding the serotonin transporter, which is related to changes in serotonin reuptake (with a role in secretion, absorption, and perception) and, secondarily, to the impairment of intestinal peristalsis [102].

The evidence for the concordance of this condition between twins is inconsistent. While some research documented a slightly greater concordance rate (2–22%) among monozygotic twins in comparison to dizygotic twins (1–9%), other studies reported concordance rates that are roughly equivalent [103,104]. In addition, one study demonstrated that a parent who has IBS is a more reliable independent predictor of the condition’s development compared to a twin sibling [105]. Thus, the familial nature of IBS is suggested, and it is believed to be a condition that could correlate with both a genetic predisposition and the social learning of harmful behaviors [105].

### 4.9. Psycho-Social Factors

**Figure 2:**
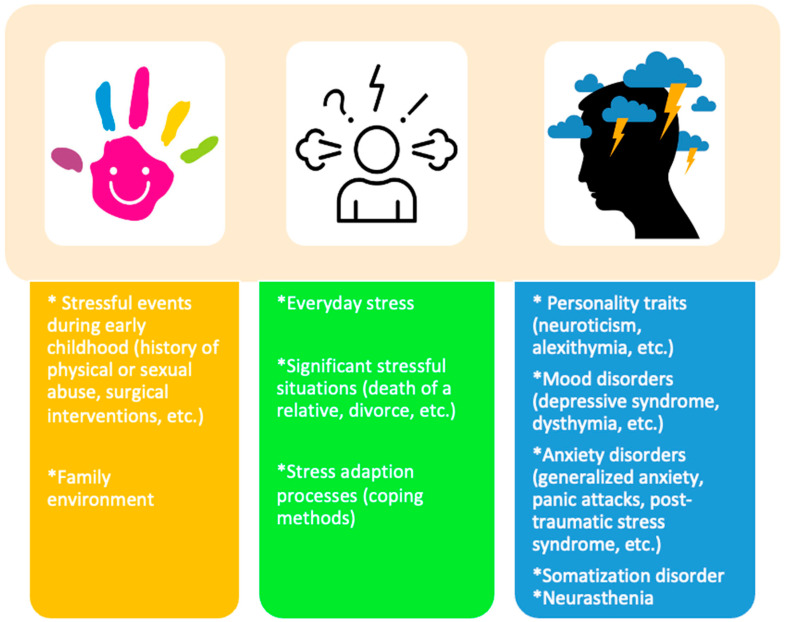
Psychological and social factors involved in the development of IBS [106,107,108,109].

The hypothesis that psychosocial factors can influence the sensory–motor function of the gastrointestinal tract appeared at the beginning of the 19th century, and much of the research evidence from that period is still valid [106]. The studies in the past few decades have delineated the biopsychosocial model, which provides an explanation for IBS [106]. All elements implicated in the pathogenesis and clinical manifestation of functional intestinal disorders are incorporated into this model [106]. Figure 2 illustrates the psychological and social factors implicated in the development of IBS [106,107,108,109,110]. They contribute to the dysregulation of the gut–brain axis, which consequently results in the development and maintenance of digestive symptoms [106].

Psychological stress is an important factor in the development of IBS [108]. The evidence from clinical and experimental studies showed a correlation between the individual’s stress level and intestinal sensitivity, motility, secretion, and permeability [108]. Among the mechanisms that can explain this pathogenic link are the activation of local immunity, changes at the level of the central and peripheral nervous systems, as well as changes at the level of the intestinal microbiota [108]. To summarize, stress can lead to changes in the microbiota-gut–brain axis, which can then result in the exacerbation or intensification of symptoms in individuals with IBS [108].

Anxiety or depression are reported by approximately one-third of patients with IBS [109]. A bidirectional relationship between psychological comorbidities and gastrointestinal symptoms is supported by data from the specialized literature [109]. A systematic review revealed that individuals with depression are at twice the risk of developing IBS in comparison to those without depression [111]. Furthermore, there is evidence to suggest that increased psychological distress is associated with a greater severity of gastrointestinal symptoms [109].

The pathogenic connection can be attributed to corticotropin-releasing factor (CRF), a peptide produced in the paraventricular nuclei of the hypothalamus and recognized as a significant modulator of the stress response [112,113,114,115,116]. Brain-level CRF hyperactivity and the CRF receptor signaling system may be associated with anxiety or depressive disorders according to some evidence [112,113,114,115,116]. Research comparing healthy subjects and patients with IBS showed that intravenous administration of CRF results in more pronounced improvements in intestinal motility and abdominal pain sensitivity in the IBS group [112]. Moreover, the administration of a CRF receptor antagonist can suppress this response without affecting the hypothalamic–pituitary–adrenal axis [112].

## 5. Differential Diagnosis of IBS

The differential diagnoses of IBS include both organic diseases and other DGBIs, which are listed in Table 3.

## 6. Discussion

To summarize, IBS is a disorder with a complex and still incompletely understood etiopathogenesis. Even if patients with IBS are not at risk of developing life-threatening complications, their quality of life is significantly affected. Another consideration is the significant cost burden that this disease imposes on healthcare systems. Chronic symptoms result in multiple costly investigations. Conversely, patients who suffer from IBS are typically young, and the existence of this ailment is linked to frequent absences from work and decreased efficiency. In light of these factors, recent research has focused on clarifying the etiopathogenesis of IBS. The main therapeutic goal is achieved through the simultaneous enhancement of patients’ quality of life and the mitigation of expenses associated with this disease. As of the present time, a sequence of pathogenic connections with a distinct pathogenic effect has been discovered. The aforementioned elements include the gut–brain axis, visceral hypersensitivity, gastrointestinal dysmotility, gut microbiota dysbiosis, food allergies and intolerances, low-grade mucosal inflammation, genetic variables, and psycho-social factors. Despite a multitude of comprehensive and complex research studies, the findings remain conflicting, and the definitive therapeutic approach for these individuals remains ambiguous. Further research involving larger cohorts of patients is required in order to clarify the pathogenic mechanisms underlying the symptoms exhibited by individuals with IBS. Furthermore, it is necessary to ascertain whether the rising prevalence of depression and anxiety disorders, as well as their frequent co-occurrence with IBS, can be attributed to the heightened vigilance exhibited by these individuals or to shared pathogenic mechanisms. It is vital to focus on the alterations in the gut microbiota, the food habits of these patients, as well as the humoral and neurological mechanisms behind their visceral hypersensitivity.

The limitation of our review is the absence of statistical analyses of the data processing procedure. Nevertheless, the narrative character of this article does not require statistical data processing.

## 7. Conclusions

To conclude, IBS is a condition with a complex pathogenesis that is still incompletely understood. The high prevalence, significant impact on the quality of life of patients, and high costs associated with this condition warrant the necessity of future research to elucidate the etiology of IBS in order to enhance the management of these patients.

## Figures and Tables

**Table 1:** ROME IV criteria for the diagnosis and classification of IBS [5].

ROME IV Diagnostic Criteria for Irritable Bowel Syndrome Abdominal pain has been present at least once a week for the past 3 months and is associated with two or more of the following: 1. In association with defecation; 2. Associated with a change in stool frequency; 3. Associated with a change in stool consistency. Criteria must be fulfilled within the last three months, with symptoms beginning at least six months before diagnosis.
Classification of Irritable Bowel Syndrome 1. Constipation-predominant IBS (IBS-C) >25% of stools have increased consistency (Bristol Scale types 1 and 2). <25% of stools have low consistency (Bristol Scale types 6 and 7). 2. Diarrhea-predominant IBS (IBS-D) >25% of stools have low consistency (Bristol Scales 6 and 7). <25% of stools have increased consistency (Bristol Scale types 1 and 2). 3. Mixed-type IBS (IBS-M) >25% of stools have low consistency (Bristol Scale types 6 and 7). >25% of stools have increased consistency (Bristol Scale types 1 and 2). 4. Non-specific IBS Modifications to the stool consistency do not allow for classification into any of the above classes.

**Table 2:** The correlation between food intolerances and IBS.

Food Allergy or Intolerance	Effects
FODMAPs	Elevation in intestinal permeability [74,75]; Low grade mucosal inflammation [74,75]; Osmotic activity [76]; Increase in short-chain fatty acid formation, intestinal gas generation, and exacerbation of luminal distention [76]; A low FODMAP diet also acts by activating Meissner’s plexus and modulating neuroenteric sensory transmission, which stimulates intestinal secretion and motility and accelerates transit time [74,75,76].
Lactose or fructose malabsorption	Abdominal flatulence [75,76,77,78].
Non-celiac gluten sensitivity	Elevation in intestinal permeability and frequency of gastrointestinal movements [79,80,81,82,83].

**Table 3:** Differential diagnoses of IBS [117].

Organic Diseases	DGBIs
Celiac disease Inflammatory bowel diseases Microscopic colitis Exocrine pancreatic insufficiency Colorectal cancer Neuroendocrine tumors Hyperthyroidism or hypothyroidism Carbohydrate malabsorption Bile acid diarrhea Small intestinal bacterial overgrowth Connective tissue disorders	Functional constipation (slow transit constipation and outlet dysfunction) Functional diarrhea Functional abdominal bloating and distension Centrally mediated abdominal pain syndrome Unspecified functional bowel disorder
